# Sustaining eSports Industry and Regulatory Focus: Empirical Evidence From Chinese Universities

**DOI:** 10.3389/fpsyg.2022.907050

**Published:** 2022-05-20

**Authors:** Gongyan Zhao, Yue Cheng, Xingguo Liu, Wentao Meng

**Affiliations:** ^1^School of Physical Education and Sports, Henan University, Kaifeng, China; ^2^Youth League Committee, Kaifeng University, Kaifeng, China; ^3^College of Education & Liberal Arts Graduate School, Adamson University, Manila, Philippines; ^4^College of Graduate Studies & Teacher Education Research, Philippine Normal University, Manila, Philippines; ^5^Institute of Higher Education, Hebei University of Science and Technology, Shijiazhuang, China; ^6^Foundation Teaching Department, Guilin University of Electronic Technology Beihai, Guangxi, China

**Keywords:** sustainability, regulatory focus, eSports, universities, China, usefulness, risk, online games

## Abstract

This study examined the factors that affect the attitude and behavioral intentions toward electronic sports (eSports) among students of higher education institutions based on the technology acceptance model (TAM). The conditional impact of preventive regulatory focus was analyzed in various aspects developed on the regulatory focus theory. These aspects comprised of perceived usefulness, perceived ease of use, and perceived risk on the attitude toward eSports. Accordingly, data were collected from 293 students of higher education institutions in China's Henan Province, presenting a 54.56% response rate. The PLS-SEM analysis was subsequently implemented to confirm the proposed hypotheses. The empirical findings confirmed the significant positive impact of perceived usefulness and perceived ease of use on the attitude toward eSports. However, perceived risk negatively affected the attitude toward eSports. Meanwhile, the findings on the moderating hypotheses found a negligible impact on preventive regulatory focus. This impact was found explicitly on the perceived usefulness-perceived ease of use link with attitude toward eSports. However, the preventive regulatory focus negatively moderated the perceived risk attitude toward eSports. Finally, the implication and limitations were illustrated at the end of the paper.

## Introduction

Higher education has undergone extensive modifications due to technological innovation (Teo et al., 2019) in integrating online information and communication, which is critical in enhancing the students' learning experience (Iqbal and Ahmad, 2021). This situation provides a conducive environment for electronic sports (eSports), which presents numerous benefits to students, i.e., enhanced critical thinking, teamwork, and self-direct learning (Wang et al., 2017). In 2022, the eSports industry is anticipated to be worth $1.79 billion, exhibiting a massive potential to be incorporated as a part of the future Olympic games (Delello et al., 2021). Notably, eSports' influence has gained traction among policymakers, academicians, and students (Rambe and Bere, 2013; Teo et al., 2019).

Due to the severe competition in the higher education industry, universities are pressured in introducing new methods and approaches to enhance the students' learning experience (Li et al., 2020). Along with this line, students will feel advantageous in learning about the presence of eSports in their institutions (Iqbal and Piwowar-Sulej, 2022). The eSports is the fastest emerging industry in educational institutions, encompassing millions of global spectators and players, where students make up the majority of them. In the United States (US), for instance, various universities are joining the National Association of Collegiate eSports (NACE) (Delello et al., 2021). Meanwhile, 600 universities have already developed 1,600 eSports clubs on their premises, though experts and analysts expect this number to grow exponentially in the coming years (McGrath, 2019).

The eSports are not only about gaming and killing time, but it also adds to the student's critical thinking skills and self-direct learning, fostering innovation and teamwork (Scott et al., 2021). Given these points, this study has considered the significance of eSports in universities and its growing industry. This study aims to enrich insights by collecting data from students on eSports in China's universities.

## Top Esports in China

Asia is experiencing massive growth in eSports, with millions watching and playing. On a global scale, China has the second biggest eSport market next to the US (Li et al., 2020). In Asia, China and South Korea are emerging as the fastest-growing eSports market. However, China is unique because players are limited to local streaming sites and do not have access to international streaming platforms (Zhao and Lin, 2021). The eSport is available in different game genres, such as first-person shooter games, real-time strategy (RTS) games, fighting games, multiplayer online battle arena (MOBA), and card battle games (Zhao and Lin, 2021). In China, players prefer shooter games, and most participate in strategy-based games in South Korea.

Moreover, the size of China's eSport industry is more significant in South Korea in terms of the number of players at the international- and national-level tournament (Scott et al., 2021). It is anticipated that the market size of China's eSport will reach 215 billion yuan in 2022. Hence, this study aims to target China's eSport industry based on the significant potential and market size. Dota 2 is the most preferred eSport game next to Overwatch and Counterstrike, played at the team level in China. However, Overwatch is the most prominent game at the individual level, followed by Dota 2 and Heroes of the Storm. Given these points, this study will be conducted for the eSport games at the individual level.

## Research Questions and Organizations

Considerable research has been conducted in traditional sports (Xiao, 2020); however, there is scant literature on eSports because of its short history (Yoshida et al., 2014). Moreover, eSports has attained adequate qualitative research (Taylor, 2016; Brock, 2017), where previous research empirically examined the player's behavior from theoretical perspectives. The theories comprise the users and gratification (Lucas and Sherry, 2004), self-determination (Przybylski et al., 2010), and the technology acceptance model (TAM) (Hsu and Chiu, 2004). The eSports and traditional sports require players to hone their skills by undergoing rigorous training sessions (Kalelo-Phiri and Brown, 2017). Past studies have emphasized the role of collaboration (Williams et al., 2021), experience-based tacit knowledge (Pizzo et al., 2022), and educational governing body (DeArmond et al., 2022) in the success of the eSports industry. Based on excitement, physical attractiveness, vicarious achievement, and family boding, eSports is unique from traditional sports, and their spectators follow different motives (Pizzo et al., 2018). The extant literature has also claimed that socialization, immersion gamification (Qian et al., 2022), skill improvement, vicarious sensation (Qian et al., 2020), the presence of star players, team loyalty, flow experiences, and self-congruity with event image (Thompson et al., 2022) positively affect spectators attendance of live streaming of eSports. On the other side, online spectators rate drama, acquisition of knowledge, appreciation of skill, novelty, aesthetics, and enjoyment of aggression consider more important than live attendees. The vicarious achievement and the novelty positively influence eSports recommendations to others (Sjöblom et al., 2020). The eSports gameplay does not influence engagement in live eSports streaming for consumers who play games frequently (Jang et al., 2021). The marketing activities in eSports events positively affect the cross-product, cross-region, and cross-firm spillover effects (Parshakov et al., 2020). The eSports players in China treat themselves as self-enterprising subjects because of meritocracy, precarity, and disposability (Lin and Zhao, 2020). However, eSports players possess the discretion to control their virtual role and stand vicariously against opponents in a virtual arena (Xiao, 2020). Therefore, it is evident that the motivation behind these two sports might be different. Thus, it is theoretically critical to enrich the eSports psychology literature by understanding the players' motives and factors for playing online games (Pizzo et al., 2018).

This study applies the TAM as the theoretical framework to examine factors related to eSports in China's universities. Primarily, researchers utilized the TAM to elaborate on the user behaviors and quantitatively applied the model in their research, such as website use (Porter and Donthu, 2006) and SME advertising (Dix et al., 2017). Others include online banking (Um, 2019), games (Ramírez-Correa et al., 2019), and shopping (Pavlou, 2003). Accordingly, extant literature suggested that the TAM is the most appropriate to explain user behaviors related to the technology (Luna-Nevarez and Torres, 2015). Therefore, in the context of eSports, TAM would effectively facilitate a practical approach to elucidating the students' behavior. This model is applied to anticipate antecedents of attitude toward eSports by treating eSports websites as constituents of technological progress.

Davis (1989) attempted to explain user behavior related to technology acceptance or rejection under the TAM. The author suggested two fundamental factors that substantially alter user attitudes toward technology-driven applications, namely, perceived usefulness and perceived ease of use. Perceived ease of use is defined as the degree to which users think that usage of a particular system is free of effort. Meanwhile, perceived usefulness concerns the impact of a particular system on the users' job performance. The two factors predict user attitudes toward the technology that drives applications and affect their behavioral intentions in technological usage (Um, 2019).

Nevertheless, the TAM only explains the user criteria in technology utilization; thus, an in-depth analysis of social factors such as perceived risk must be implemented to obtain a clear picture of user behavior (Shen and Eder, 2011; Um, 2019). Hence, the present research integrates perceived risk into the TAM as an additional eSports driver among students (Shen and Eder, 2011; Weisberg et al., 2011), which is due to the apparent differences between traditional sports and eSports. Given these points, this study aims to examine the impact of perceived usefulness, perceived ease of use, and perceived risk on the attitude toward eSports. Finally, the current research assesses the effect of attitude toward eSports on the behavioral intentions toward eSports.

The eSport users are expected to elicit negative responses because of privacy concerns involved while playing (Zarouali et al., 2019). Researchers claimed different responses from various users, albeit there is less evidence about their potential behaviors (Zarouali et al., 2017). There are no comprehensive studies to expose how individual differences between users alter the variations in their responses to eSports at the individual level. Thus, this study aims to examine how motivation differences between the students influence their attitude toward eSports. The regulatory focus theory (Higgins, 2002) is employed, where users adopt different motivational orientations while pursuing their goals. This theory concerns the extent to which an individual is motivated to avoid hazards or realize achievements (Zhao and Pechmann, 2007).

Therefore, this study investigates relevant theoretical backbone in assessing students' different behavior in eSports, which involve privacy intrusions. The moderating impact of preventive regulatory focus is determined based on the relationships of perceived ease of use, perceived usefulness, and perceived risk with attitude toward eSports among students in China's universities. Meanwhile, several contributions were made to the theory and eSports-related literature. First, eSports emerged as the top trend in China in just a few years and is viewed as the main subject among academicians and practitioners. This study has enriched literature by exploring antecedents of eSports behavior among students in Chinese universities. Second, the study provides insights into students' behaviors toward eSports by delivering empirical evidence. Third, the theory of validating the proposed framework is enriched, especially on the TAM. Finally, the authors contributed to the fields of games from the business and social perspectives. Hence, eSports enhance specific skills among students; thus, universities can design their eSports environment based on current findings.

## Hypotheses Development

The perceived usefulness is the extent to which users believe that an application positively influences their performance (Davis, 1989). It was found that significant perceived usefulness strengthens the relationship of use with performance (Przybylski et al., 2010). Past technologies have confirmed the positive impact of perceived usefulness, fostering a positive attitude toward technologies (Um, 2019). In online gaming, performance expectancy significantly influences behavioral intentions (Ramírez-Correa et al., 2019). In the mobile industry, the advertisement's perceived usefulness is positively related to the user attitude toward mobile advertising (Shankar and Datta, 2018). A similar phenomenon can be observed in the user attitude toward social networking advertisements (Luna-Nevarez and Torres, 2015) and e-commerce (Yeo et al., 2017). Based on the above discussion, the following hypothesis is proposed.

*H1: Perceived usefulness is positively related to the attitude toward eSports*.

The perceived ease of use concerns how effortless a particular system feels to a person (Davis, 1989). Users are more likely to use an application or new technology if they perceive effortlessness in its usage (Chen and Aklikokou, 2020; Poulus et al., 2020). In eSports, effort expectancy is directly related to online games (Ramírez-Correa et al., 2019). Moreover, past studies reported that perceived ease of use positively impacts user attitudes toward e-commerce websites (Ashraf et al., 2016; Abdullah et al., 2017). Hence, the following hypothesis is postulated:

*H2: Perceived ease of use is positively related to the attitude toward eSports*.

At present, eSports is a multi-million-dollar industry (Gough, 2021; Keller et al., 2021), where risk-taking leads to significant rewards. For instance, a recent eSports tournament has offered more than 35 million dollars in prizes, which is more significant than the Wimbledon tournament winner (Rudolf et al., 2020). Notably, eSports players are not subjected to physical risks, albeit it is still crucial to assess the risk inclination (Keller et al., 2021). Nevertheless, there is scant research on the risk behavior in this industry (Pedraza-Ramirez et al., 2020). Past studies reported a significant association between perceived privacy with user intention (Yu and Song, 2021). For instance, it was found that the privacy risk among online users influences their intention through user trust level (Dinev and Hart, 2006). Meanwhile, a study on social media users reported that higher perceived risk is inversely related to their risk-taking propensity (Wang and Lin, 2017).

A study among local-based service users found that perceived is negatively related to perceived trust (Wang and Lin, 2017). Furthermore, perceived risk was found to negatively affect users' risk-taking intentions (Yu and Song, 2021). The shift in the risk-taking phenomenon revealed that privacy concerns negatively influence the intention to use LBS services (Ramírez-Correa et al., 2019). Therefore, the following hypothesis is developed:

*H3: Perceived risk is negatively related to the eSport players' attitude*.

Previous literature implemented various theories such as the theory of planned behavior, theory of reasoned action, the TAM, and repurchase decision-making theory (Davis, 1989; Hwang et al., 2019; Chen and Aklikokou, 2020). Studies confirmed the critical role of attitude in developing behavioral intentions (Fu and Elliott, 2013; Han et al., 2017). For instance, Hung et al. (2006) found that attitude affected the public's behavioral intentions to accept e-government services using 1,099 usable responses. Additionally, Fu and Elliott (2013) collected data from 312 customers who purchased a new technology to identify their relationship attitudes and behavioral intentions. The analysis revealed that customers are more likely to utilize the product when they have a favorable attitude toward using new technologies.

Another study examined how behavioral intentions are enhanced in the online shopping industry using a sample of 2,631 consumers (Wu and Ke, 2015). They suggested that customers tend to show affirmative behavioral intentions when they have a positive attitude. A similar finding is found in 429 tourists in an environmentally responsible museum (Han et al., 2017), indicating that attitude enhances behavioral intentions. Meanwhile, Munoz-Leiva et al. (2017) analyzed 103 regular electronic banking users, revealing attitude as an essential factor affecting behavioral intention. Given the theoretical and empirical findings, it is expected that attitude toward eSport positively impacts behavioral intention.

*H3: Attitude is positively related to behavioral intentions toward eSports*.

The regulatory focus theory significantly influences user behavior due to its ability to express various psychological processes and behaviors (Higgins, 1997; Haws et al., 2010). An essential part of this theory considers the evaluation of distinct user-related stimuli. Under prevention focus, information related to utilitarian and necessary features carries a more significant weight (Hassenzahl et al., 2008; Roy and Ng, 2012). For instance, it was found that prevention-focused consumers favor the safety and protection dimension of a car rather than the accomplishment of its dimensions (Safer, 1998). Correspondingly, individuals favor prevention rather than promotion focus, especially in selecting sun lotions that stress the importance of skin protection (safety dimension) (Florack et al., 2006). Other studies found similar results; prevention-focused consumers evaluate stimuli more favorably when perceiving risk prevention (Zarouali et al., 2019). In other words, prevention-focused consumers prefer to preserve the status quo, thereby protecting their safety and avoiding risks.

Generally, consumers are concerned with safety and privacy protection features; thus, this idea should carry a greater weight under a prevention focus (Wirtz and Lwin, 2009). Consumers tend to focus on the most relevant dimensions, especially in evaluating the regulatory focus stimuli, such as products or advertisements (Florack et al., 2006; Roy and Ng, 2012). In line with these findings, adolescents are expected to exhibit different dimensions or goals when confronted with targeted advertising. However, they should perceive it as a privacy intrusion as their personal information is collected and used for an inappropriate persuasion attempt. Based on this reasoning, we expect prevention-oriented adolescents to present a negative attitude toward targeted advertising. Thus, the following hypotheses are postulated:

*H4: Perceived usefulness-attitude toward eSports relationships is diminished with the presence of a higher preventive regulatory focus*.
*H5: Perceived ease of use -attitude toward eSports relationships is diminished with the presence of a higher preventive regulatory focus*.
*H6: Perceived risk-attitude toward eSports relationships is strengthened with the presence of a higher preventive regulatory focus*.

## Research Methodology

China is experiencing rapid growth in its eSports industry next to South Korea, though, unlike the latter, its industry is unique in terms of its size and access to the local software and applications. Due to these distinctive features, the current research selected players from China as the study's population. Due to the time and financial constraints, purposive sampling was employed based on the criteria of whether a student has participated in eSports at least once. Next, the data were collected from students of public and private universities in the Henan Province of China. An online questionnaire was constructed comprising seven sections: perceived usefulness, perceived ease of use, perceived risk, attitude toward eSports, behavior toward eSports, preventive regulatory focus, and demographic information.

The online survey form was shared through social media applications such as WeChat, QQ, and a survey platform (WJX), in which 537 questionnaires were received. However, there are only 293 valid forms for further analysis after the screening, indicating a recovery rate of 54.56%. Notably, the current sample size is more significant than 10 times the largest structural paths in the proposed framework (Hertzog, 2008). Hence, this sample size is sufficient to conduct the partial-least structural equation modeling (PLS-SEM) analysis.

### Measures

The measurement scales of all continuous variables in this study were adopted from the extant literature, albeit slight modification was done concerning eSports. The data collection was based on a five-point Likert scale, ranging from strongly agree to strongly disagree. Accordingly, two to four items were utilized to evaluate the perceived usefulness (Pavlou, 2003), perceived ease of use (Venkatesh and Davis, 1996), perceived risk (Pavlou, 2003), attitude toward eSports (Davis, 1989), and behavioral intentions toward eSports (Rudolf et al., 2020). Meanwhile, the chronic self-regulatory focus was assessed using the general regulatory focus measure (Lockwood et al., 2002), comprising nine items. These items measure prevention focus, i.e., “In general, I am focused on preventing negative events in my life,” in which the response categories ranged from one (strongly disagree) to five (strongly agree).

### Demographic Analysis

The demographic analysis showed that male participants (*n* = 217, 78.34%) dominated in this investigation compared to women (*n* = 60, 21.66%). Most participants were aged 16–20 years (*n* = 141, 50.90%), followed by those (*n* = 107, 38.63%) who fall in the age bracket of 21–25 years. The most significant participation originated from students in public universities (*n* = 169, 61.01%), whereas the least number is from private universities.

### Data Screening

Data screening must be conducted before data analysis, revealing missing values, outliers, data normality, and common method bias. Thus, this study employed online survey forms to collect data and marked mandatory checks against every item. The dataset is freer of any outliers, provided the *Z*-score values are <3.29 (Tabachnick et al., 2007). We deleted four items to ensure the absence of outliers in the data, as their values were more significant than 3.29. Furthermore, this study examined all continuous variables' skewness and kurtosis values to determine the data normality. The skewness values of all continuous variables lie between −0.428 and +1.382 (refer to Table 1). Meanwhile, the kurtosis values fall between −0.574 and 7.405 (refer to Table 1), out of the 3 range (DeCarlo, 1997). These items include perceived usefulness, perceived ease of use, perceived risk, attitude, behavioral intentions, and preventive regulatory focus. Hence, the dataset in this study is non-normal.

**Table 1:** Descriptive analysis.

**Construct**	**Mean**	**Std. deviation**	**Skewness**		**Kurtosis**	
	**Statistic**	**Statistic**	**Statistic**	**Std. error**	**Statistic**	**Std. error**
Preventive regulatory focus	4.217	0.550	−0.249	0.146	−0.574	0.292
eSports behavior	2.977	0.634	0.787	0.146	6.486	0.292
Attitude toward eSports	3.669	0.865	1.382	0.146	7.405	0.292
Perceived usefulness	4.008	1.088	0.335	0.146	4.317	0.292
Perceived ease of use	4.048	1.007	0.485	0.146	5.361	0.292
Perceived risk	4.294	0.584	−0.428	0.146	−0.508	0.292

Nevertheless, the PLS-SEM does necessitate data normality. The data collected from a single source may raise biasness in the empirical findings; thus, several methods must be implemented to check for potential biasness in the dataset. These methods comprise Harman's single factor test (Jarvis et al., 2003), marker variables (Simmering et al., 2015), and correlation matrix procedures (Esposito Vinzi et al., 2010). Currently, researchers have suggested the marker variables approach because of its higher validity and reliability (Hair et al., 2020). Moreover, the attitude was regressed against each independent variable to examine the common method bias, namely, perceived ease of use, perceived usefulness, perceived risk, and preventive regulatory focus. Malhotra et al.'s (2017) criteria consider the change in R-squared values. Notably, the change in *R*-squared is <10% for the inclusion of attitude; hence, this study is freer from any biasness.

### Descriptive Statistics

All continuous variables are measured based on a five-point Likert scale. However, there is a low, moderate, and high presence of any variable measured on this scale, given that its mean value is ≤ 2.99, between 3 and 3.99, and >4/00 (Sekaran and Bougie, 2016). Meanwhile, the mean values of perceived usefulness (M = 4.008), perceived ease of use (M = 4.048), perceived risk (M = 4.294), and preventive regulatory focus (M = 4.217) are above 3.99 (refer to Table 1). Therefore, this analysis reveals high-level practices of these variables among the students related to eSports, though their attitude toward eSports is at a moderate level (M = 3.669). However, the descriptive analysis revealed students' shallow behavioral usage intentions (M = 2.977) toward eSports.

### Measurement Model Analysis

The position of reliability and validity of the constructs and indicators were determined using the measurement model analysis by assessing factor loadings, composite reliability, and Cronbach's alpha. An indicator presents acceptable reliability if the factor loading value exceeds 0.50 (Chin, 1998). Regardless, any value below 0.40 is acceptable if the average variance extracted (AVE) value is >0.50 (Hair et al., 2020). Table 2 shows that all items exhibited values >0.50, signifying acceptable indicator reliability. Subsequently, internal consistency reliability is evaluated based on Cronbach's alpha and composite reliability (CR), presenting acceptability if both values are more significant than 0.70.

**Table 2:** Factor loadings, composite reliability, and AVE values.

**Constructs**	**Items**	**Attitude toward eSports**	**Cronbach's Alpha**	**Composite Reliability**	**Average Variance Extracted**
Attitude toward eSports	AE1	0.876	0.820	0.880	0.656
	AE2	0.930			
	AE3	0.522			
	AE4	0.847			
Behavioral intentions to eSports	BI1	0.892	0.894	0.934	0.825
	BI2	0.911			
	BI3	0.921			
Perceived ease of use	EOU1	0.947	0.936	0.953	0.836
	EOU2	0.950			
	EOU3	0.934			
	EOU4	0.820			
Perceived risk	PR1	0.852	0.739	0.882	0.790
	PR2	0.924			
Preventive regulatory focus	PRF1	0.710	0.925	0.937	0.624
	PRF2	0.704			
	PRF3	0.825			
	PRF4	0.765			
	PRF5	0.804			
	PRF6	0.830			
	PRF7	0.823			
	PRF8	0.781			
	PRF9	0.853			
Perceived usefulness	PU1	0.972	0.956	0.972	0.920
	PU2	0.968			
	PU3	0.937			

The Cronbach's alpha values of various items presented >0.70, i. e, perceived ease of use (α = 0.936), perceived usefulness (α = 0.956), and perceived risk (α = 0.739). Others include preventive regulatory focus (α = 0.925), attitude toward eSports (α = 0.820), and behavioral usage toward eSports (α = 0.894) (refer to Table 2). However, the CR values of continuous variables in this study are between 0.880 and 0.972, which does not consider values below 0.70 (refer to Table 2). Thus, all continuous variables in this study have acceptable internal consistency.

Construct validity revolves around convergent and discriminant validity, requiring factor loadings values >0.40 and AVE >0.50 (Hair et al., 2017). This study reported that various items exhibit AVE values >0.50, implying acceptable convergent validity. The items comprise perceived ease of use (AVE = 0.836), perceived usefulness (AVE = 0.920), and perceived risk (α = 0.790). Other items include preventive regulatory focus (α = 0.624), attitude toward eSports (α = 0.656), and behavioral usage toward eSports (α = 0.825) (refer to Table 2).

The Fornell–Larcker criterion and heterotrait–monotrait ratio (HTMT) were employed to assess the discriminant validity of the constructs. The Fornell–Larcker criterion confirms discriminant validity if the constructs' square root of AVE values is more significant than the inter-construct correlation values (Fornell and Larcker, 1981). Table 3 reveals that the square root of the AVE of all constructs is more significant than their inter-construct correlations values, implying acceptable discriminant validity. Similarly, the heterotrait–monotrait ratio (HTMT) proved discriminant validity of all constructs as all values were found below 0.85, requiring values of all ratios below 0.85 (Kline, 2015).

**Table 3:** Fornell–Larcker criterion.

**Constructs**	**1**	**2**	**3**	**4**	**5**	**6**
Attitude toward eSports	0.810					
Behavioral intentions to eSports	0.802	0.908				
Perceived ease of use	0.358	0.379	0.914			
Perceived risk	0.495	0.500	0.361	0.889		
Perceived usefulness	0.420	0.439	0.903	0.314	0.959	
Preventive regulatory focus	0.609	0.627	0.394	0.825	0.392	0.790

### Hypothesis Testing

The structured model was assessed through 5,000 subsamples using bootstrapping method through SmartPLS. The structural model analysis reported that perceived usefulness significantly influences attitude toward eSports (β = 0.653, ρ < 0.000) (refer to Table 4), supporting hypothesis H1. Furthermore, the perceived ease of use (β = 0.433, ρ < 0.000) and perceived risk (β = −0.402, ρ = 0.029 < 0.000) significantly affect attitude toward eSports among the students. This result offered sufficient support for the acceptance of hypotheses H2 and H3. Moreover, the students' attitude toward eSports is positively related to their behavioral intentions (β = 0.871, ρ < 0.000); hence, hypothesis H4 is supported.

**Table 4:** Hypotheses testing.

**Hypothesis**	**β**	**S.D**	***T*** **-value**	***p*** **-value**	**LLCI**	**ULCI**
Perceived usefulness -> attitude toward eSports	0.653	0.181	3.618	0.000	0.282	0.979
Perceived ease of use -> attitude toward eSports	0.433	0.063	6.926	0.000	0.322	0.556
Perceived risk -> attitude toward eSports	−0.402	0.184	2.185	0.029	−0.747	−0.016
Attitude toward eSports -> behavioral intentions to eSports	0.871	0.026	33.366	0.000	0.813	0.914
Perceived usefulness * preventive regulatory focus -> attitude toward eSports	0.016	0.169	0.098	**0.922**	−0.380	0.271
Perceived ease of use * preventive regulatory focus -> attitude toward eSports	0.042	0.161	0.259	**0.796**	−0.337	0.299
Perceived risk * preventive regulatory focus -> attitude toward eSports	−0.169	0.055	3.050	0.002	−0.080	−0.289

*Bold indicates p value > 0.05*.

The moderation analysis revealed that the interaction in perceived usefulness and preventive regulatory focus does not significantly influence attitude toward eSports (β = 0.016, ρ>0.000) (refer to Table 4). Hence, preventive regulatory focus does not moderate the relationship between perceived usefulness and eSports attitude, rejecting hypothesis H5. Similarly, interaction in perceived ease of use and preventive regulatory focus does not affect eSports' attitude (β = 0.042, ρ>0.000). Preventive regulatory focus exhibited a non-significant moderating role in eSports' perceived ease of use-attitude. Therefore, H6 is not supported. Nevertheless, preventive regulatory focus significantly moderates the perceived risk-attitude toward eSports behavior (β = −0.169, ρ < 0.000) among the students (refer to Table 4), supporting hypothesis H6. This finding revealed the negative impact of perceived risk on the attitude toward eSports. Specifically, this impact becomes increasingly severe at the higher values of preventive regulatory focus.

## Discussion

The results of hypothesis H1 are parallel with previous studies (Mohammadi and Isanejad, 2018; García-Fernández et al., 2020; Weng, 2021; and Syahruddin et al., 2021). Previous findings concluded that perceived usefulness and perceived ease of use positively influence user attitudes toward various aspects. These aspects comprise online learning in higher education institutions (Syahruddin et al., 2021), information technology in sports organizations (Mohammadi and Isanejad, 2018) and fitness applications (García-Fernández et al., 2020). Another example study found that perceived usefulness positively impacts the attitude toward microblogging in China (Weng, 2021). Nevertheless, the current findings are inconsistent with previous ones (Huang et al., 2015; Sukendro et al., 2020). These studies reported that perceived usefulness exhibited a negligible effect on the attitude toward e-learning in Indonesia and GPS applications in Taiwan.

Extant literature provided comparable results vis-à-vis the perceived ease of use's positive effect on the user's attitude (Dhurup and Dlodlo, 2013; Ibrahim, 2014; Huang et al., 2015; Mohammadi and Isanejad, 2018; García-Fernández et al., 2020; Sukendro et al., 2020; Syahruddin et al., 2021). For instance, perceived ease of use significantly influenced the attitude toward e-learning in Indonesia (Sukendro et al., 2020) and fantasy sports websites in the USA (Ibrahim, 2014). Other studies revealed congruent findings, such as GPS in golf games in Taiwan (Huang et al., 2015) and fantasy football in South Africa (Dhurup and Dlodlo, 2013).

Nevertheless, other studies have negated the positive perceived ease of use-attitude relationship (Goebert and Greenhalgh, 2020; Weng, 2021). In China, a study among microbloggers found a non-significant effect of perceived ease of use on their attitude toward microblogging (Weng, 2021). Correspondingly, a similar finding was found in the case of utilizing augmented reality in the USA (Goebert and Greenhalgh, 2020). However, there are no prior studies conducted on the relationship of perceived risk with attitude toward online games. The current findings confirmed that perceived risk significantly negatively affects attitude toward eSports in China. Hence, the results of hypothesis H3 fulfilled the research gap in online games, especially in the eSports industry.

All things considered, the empirical evidence contrasts with O'Connor et al.'s (2021) findings. In this case, hypotheses H4 and H5 posited that preventive regulatory focus significantly moderates the relationship between perceived usefulness and ease of use with user attitude toward eSports. However, current findings found a non-significant relationship in this matter; thus, hypotheses H4 and H5 are not supported. The current empirical findings in moderating factors are opposite to those provided by O'Connor et al. (2021). Specifically, the perceived usefulness and ease of use significantly influence user's attitudes toward digital technology.

Based on these results, the present findings confirmed that perceived risk negatively impacts the attitude toward eSports. This impact became increasingly critical in the presence of higher preventive regulatory focus values. This discovery aligned with Zhang et al. (2018), who reported a reduced negative impact of perceived risk on the attitude toward electric vehicles. This diminished impact is due to the presence of the promotion instead of the preventive regulatory focus among consumers in China.

### Theoretical Implications

This study offers numerous theoretical and practical implications. Theoretically, this study confirms the significance of perceived ease of use, usefulness, and perceived risk in students' attitudes toward eSports, and their behavioral intentions. This research extended literature based on the technology acceptance model and demonstrated that its application would substantially alter students' attitudes toward eSports. Additionally, this study reinforces the customary notion by offering robust support regarding the impact of perceived ease of use, usefulness, and risk concerning TAM on the students' attitude and behavioral intention toward eSports in China.

Theoretically, this study also enriches literature in the field of regulatory focus theory. Extant literature has applied regulatory focus theory as a backbone to explore the issues related to the privacy of consumers (Wirtz and Lwin, 2009). No prior study has assessed the power of regulatory focus theory in explaining users' responses to the eSports industry (Zarouali et al., 2019). Regarding this, this study extended empirical shreds of evidence related to the moderating role of preventive regulatory focus on the relations of perceived usefulness, perceived ease of use, and perceived risk with students' attitude toward eSports. The extant literature offers findings on the regulatory focus in the marketing field from the perspective of adult consumers (Zarouali et al., 2019). This study evaluates its applicability in the domain of the eSports industry. Here, we claim regulatory focus as a robust framework for explaining the players' attitudes. Past studies conclude that regulatory focus may alter the consumers' responses toward a specific product. This study only focuses on the preventive nature of regulatory focus in the eSports industry.

### Practical Implications

This study offers few implications for policymakers, marketers, and management of higher education institutions. The present findings disentangled that students are ready to participate in eSports provided they find it an easy and valuable platform. Before launching any platforms in universities, instructional designers, teachers, and educational leaders should work on these internal factors such as risk, usefulness, and ease of use, which cultivate students' positive attitudes. The policymakers and management of higher education institutions need to focus on the value, complexity, and risk involved in eSports to boost students' participation in eSports events. They could invite other well-known players to share their positive and valuable experiences with eSports to alter the students' perceptions of complexity and risk level. Students' attitudes toward eSports could be highly encouraged and will exhibit positive behavioral intentions toward eSports.

The current research confirms that preventive regulatory focus significantly moderates the perceived risk-attitude toward eSports relationship among universities students in China. Thus, the present findings demonstrated that the preventive regulatory focus as an individual trait is handy in fostering students' behavior toward eSports. To target prevention-oriented students, universities should try to allure students toward eSports using minimum personal information to ensure the effectiveness of their programs. The management of universities in China should have adequate knowledge of their target students and more precise knowledge of whether potential students are mainly prevention or promotion-focused. The practitioners may use interest-based data of students to decide about their dominant regulatory focus easier. Adult role models are helpful for teens to develop their identity while growing up (Steinberg, 2010; Trotter et al., 2021). As developing their identity, teens like and follow different celebrities and role models on social media. The prevention-focused students are motivated by negative role models (Zarouali et al., 2019). Therefore, teachers and university management may find such information valuable to decide about the regulatory focus orientation of students.

## Conclusion

Sketching on the technology acceptance model (TAM) and regulatory focus theory, this study identifies the vital determinants of behavioral intentions toward eSports in higher education institutes in China. We collected data from students in the Henan province of China. This study examines the impact of perceived ease of use, usefulness, and risk on students' attitudes toward eSports. The present empirical findings confirm that the perceived ease of use and the usefulness significantly affect students' attitudes toward eSports. Yet, their perceived risk negatively influences their attitude toward eSports in China. Furthermore, this study confirms that students' attitude toward eSports positively influences their behavioral intentions. This study also examines the moderating effect of preventive regulatory focus on the relationship of perceived ease of use, usefulness, and risk with attitude toward eSports in higher education institutions in China. The current findings only claim that the negative impact of the perceived risk on the attitude toward eSports intensifies in the presence of higher values of preventive regulatory focus. This study does not offer empirical support regarding the moderating impact of preventive regulatory focus on the relationship of the perceived usefulness and ease of use on the student's attitude toward eSports.

This study presented several limitations despite having substantial theoretical and empirical contributions. First, data collected from students were limited to China's Henan province; thus, larger sample size must be utilized from different provinces to extend the generalization of the findings. Second, the data are cross-sectional, and thus, future studies should adopt a longitudinal approach, unraveling the significance of integrated relations of the variables. Third, only a single aspect of regularity focus was investigated, namely, preventive regulatory focus. Therefore, researchers must assess this relationship under the umbrella of a promotion-based regulatory focus. Accordingly, other lines of studies could examine the impact of personality traits on the attitude toward eSports and behavioral intentions.

## Data Availability Statement

The original contributions presented in the study are included in the article/supplementary material, further inquiries can be directed to the corresponding author/s.

## Author Contributions

GZ, YC, and XL conceived and designed study, collected and complied, and analyzed data. YC did statistical analyses. YC, XL, and WM drafted and edited manuscript. All authors contributed to the article and approved the submitted version.

## Funding

This work is supported by 2022 General Project of Education Science Planning in Henan Province, China under project name “Study on the Students influence mechanism of electronic sports participation behavior in Universities in Henan Province”. This work is supported by 2022 Sports research project of Henan Provincial Sports Bureau in China under project name “Research on the Influence mechanism of Electronic sports Participation behavior among College students in Henan Province”. This work is supported by 2021 National Social Science Foundation project in 2021 in China (21BSH072) under the project name “Research on community health margin and protection system construction under major epidemic”.

## Conflict of Interest

The authors declare that the research was conducted in the absence of any commercial or financial relationships that could be construed as a potential conflict of interest.

## Publisher's Note

All claims expressed in this article are solely those of the authors and do not necessarily represent those of their affiliated organizations, or those of the publisher, the editors and the reviewers. Any product that may be evaluated in this article, or claim that may be made by its manufacturer, is not guaranteed or endorsed by the publisher.
